# Workplace Gaslighting: Implications for Employees’ Mental Health and Work Life in Greece

**DOI:** 10.3390/healthcare13243255

**Published:** 2025-12-12

**Authors:** Ioannis Moisoglou, Aglaia Katsiroumpa, Olympia Konstantakopoulou, Polyxeni Mangoulia, Maria Tsiachri, Aristotelis Koinis, Georgios Marios Kyriakatis, Petros Galanis

**Affiliations:** 1 Department of Nursing, University of Thessaly, 41500 Larissa, Greece; aristokoinis@uth.gr; 2 Clinical Epidemiology Laboratory, Faculty of Nursing, National and Kapodistrian University of Athens, 11527 Athens, Greece; aglaiakat@nurs.uoa.gr (A.K.); mtsiachri@nurs.uoa.gr (M.T.); gmkyriakatis@nurs.uoa.gr (G.M.K.); pegalan@nurs.uoa.gr (P.G.); 3 Center for Health Services Management and Evaluation, Faculty of Nursing, National and Kapodistrian University of Athens, 11527 Athens, Greece; olympiak1982@hotmail.com; 4 Laboratory Nursing Counselling and Psychoeducation of Patients and Caregivers, Faculty of Nursing, National and Kapodistrian University of Athens, 11527 Athens, Greece; pmango@nurs.uoa.gr

**Keywords:** anxiety, employees, mental health, quiet quitting, work engagement, workplace gaslighting, work life

## Abstract

**Background/Objectives**: The present study seeks to address an important empirical gap by examining the associations of workplace gaslighting with symptoms of anxiety and depression, quiet quitting, and work engagement among a sample of Greek employees. **Methods**: An online cross-sectional study was conducted in Greece in December 2024, with 291 employees, aged 18 years or older, who reported at least one year of work experience. The validated Greek versions of already published tools were used to measure workplace gaslighting (GWS), anxiety and depression (PHQ-4), quiet quitting (QQS) and work Engagement (UWES-3). Associations between gaslighting and mental health and occupational outcomes were tested using multivariable linear regression, adjusting for demographic and occupational covariates. **Results**: Higher workplace gaslighting scores were significantly predictive of anxiety (b = 0.565, *p* < 0.001) and depression (b = 0.571, *p* < 0.001). Gaslighting was also a significant predictor of both quiet quitting (b = 0.368, *p* < 0.001) and work engagement (b = −0.373, *p* < 0.001). **Conclusions**: These results highlight the negative consequences of gaslighting on the mental health and work engagement of employees. Employees should be encouraged to report instances of supervisory gaslighting, while senior leadership and organizational governance structures ought to implement and enforce a zero-tolerance policy toward such behaviors.

## 1. Introduction

Effectiveness and efficiency lie at the core of organizational management worldwide, regardless of the field of activity or ownership status (public or private). Undeniably, performance is closely linked to the survival of organizations and represents the primary outcome within their strategic planning framework. The most valuable resource supporting this endeavor is the workforce, which is responsible for managing all other resources and achieving the organization’s goals. However, this effort is far from a smooth journey paved with ease and comfort. Heavy workloads, understaffing, overtime, and fatigue often characterize employees’ working environments [1,2,3]. At the same time, mental health issues such as stress, anxiety, burnout, depression, and sleep disturbances are common concerns among workers [4,5,6]. The challenging work environment contributes to a reduction in work engagement, leading employees either to change their job positions or to remain in their current roles while opting for quiet quitting [7,8,9].

In employees’ efforts to achieve organizational goals, enhance productivity, and foster a work environment characterized by occupational well-being, the role of supervisors and the leadership style they adopt is considered fundamental. When the supervisor adopts a transformational leadership style, they succeed in motivating employees, fostering innovation and creativity, and contributing to improved performance [10,11,12]. Supervisors who inspire their employees, stimulate them to engage in creative thinking, and take into account each follower’s individual needs, thereby ensuring their well-being and psychological functioning [13]. Through empowering, strengthening, and connecting behaviors, supervisors enhance employees’ work engagement and reduce the likelihood of quiet quitting [14]. Hence, a supportive supervisory role contributes positively to employees’ workplace behavior and overall performance.

Despite the positive effects of supportive leadership on employees, they often experience abusive behavior, leading to adverse consequences for both themselves and their employing organizations. Such behavior includes gaslighting, a form of psychological manipulation and abuse in which the perpetrator (gaslighter) seeks to instill doubt in the victim regarding their cognitive functioning (e.g., memory, judgment), ultimately leaving the individual confused, disoriented, and vulnerable [15]. In this endeavor, the gaslighter employs techniques such as denial (refusing to acknowledge truths despite clear evidence and replacing meaningful dialogue with dismissive statements), deception, dismissal, minimization, behavioral inconsistency, isolation, and coercion [16]. Such behavior may originate from the gaslighter’s underlying insecurity, a strong need for self-validation and dominance, a tendency to suppress dissenting perspectives, and an unconscious attempt to mitigate personal anxiety through mechanisms of projective identification [16,17,18]. Other factors that may trigger the development of gaslighting behaviors include gendered stereotypes, structural vulnerabilities related to race, nationality, and sexuality, and institutional inequalities [19].

The consequences of gaslighting on victims’ mental health include impaired psychological functioning, anxiety, Post-Traumatic Stress Disorder (PTSD), guilt, depression, and grief, as well as suicidal ideation and behavior [20]. Similar effects of gaslighting are observed among employees, with victims being more likely to experience depression, anxiety, and burnout [21,22]. In organizations where supervisors engage in gaslighting behaviors, employees report higher levels of turnover intention, along with reduced affective organizational commitment, motivation, and job embeddedness [21,23,24]. In interpersonal relationships, gaslighting victimization has been linked to a weakened sense of self, increased distrust toward others, and, in some cases, experiences of post-traumatic growth [25].

Abusive leadership behavior refers to a sustained pattern of hostile verbal and nonverbal actions by leaders toward subordinates, excluding physical aggression. This phenomenon typically occurs within hierarchical organizational structures where power asymmetry enables supervisors to exert control through intimidation, ridicule, or deliberate disregard for employees’ contributions. Such behaviors are not isolated incidents but persistent interactions that undermine psychological safety and erode trust within the workplace [26]. Abusive leadership is associated with detrimental outcomes, including increased employee stress, reduced job satisfaction, and higher turnover intentions, ultimately impairing organizational performance [27]. Employees who are subjected to abusive supervision experience resource depletion and may respond through withdrawal or counterproductive work behaviors [28]. Within this framework of abusive leadership behavior, the aim of the present study was to investigate the impact of gaslighting on employees’ mental health, quiet quitting and work engagement.

## 2. Materials and Methods

### 2.1. Study Design

A cross-sectional study was carried out in Greece using an online survey distributed in January 2025. The questionnaire was developed in Google Forms and shared in Facebook and Instagram groups dedicated to workers, as well as sent directly to workers via LinkedIn messages. In particular, we created an introductory note to inform participants about the aim and the design of the study. The introductory note included the inclusion and exclusion criteria as follows: (a) employees who had worked at least one year can participate in this study, (b) supervisors or managers cannot participate in this study since we measure their attitudes towards employees, (c) all employees independently the level of workplace gaslighting they suffer can participate in this study, (d) all employees regardless their mental health status can participate in this study, (e) employees from any organization or service who report to a supervisor can participate in this study, (f) white-collar workers perform administrative, managerial, or knowledge-based work, typically in an office setting can participate in this study, and (f) employees who give their informed consent can participate in this study. This approach resulted in a convenience sample of employees in different units, workplaces and institutions. There were no incentives for participation in the study. The study followed the “Strengthening the Reporting of Observational Studies in Epidemiology” (STROBE) guidelines [29]. The study examined workplace gaslighting as the main predictor, with gender, age, educational attainment, and work experience considered as confounding variables. Anticipating an effect size of 0.04 between workplace gaslighting and each outcome (anxiety, depression, quiet quitting, and work engagement), with a statistical power of 95% and a 5% margin of error, the required sample size was calculated to be 262 workers using G*Power v.3.1.9.2.

### 2.2. Measurements

Workplace gaslighting was assessed using the Gaslighting at Work Scale (GWS), which contains 11 items covering two domains: “loss of self-trust” (five items) and “abuse of power” (six items) [30]. Responses are rated on a five-point Likert scale from 1 (never) to 5 (always), with higher scores indicating more frequent gaslighting behaviors. The Greek version of the GWS was used, showing high reliability (Cronbach’s alpha for the total scale: 0.945; “loss of self-trust”: 0.918; “abuse of power”: 0.909).

Anxiety and depression were measured using the Patient Health Questionnaire-4 (PHQ-4), which includes four items, two for anxiety and two for depression, answered on a four-point Likert scale (0 = not at all to 3 = nearly every day) [31]. Higher scores reflect greater symptom severity. The Greek version was used, with Cronbach’s alpha values of 0.840 (anxiety) and 0.862 (depression) [32]. Quiet quitting was evaluated with the Quiet Quitting Scale (QQS), a nine-item instrument with three subscales: “detachment” (four items), “lack of initiative” (three items), and “lack of motivation” (two items) [33]. Responses are on a five-point Likert scale from 1 (strongly disagree/never) to 5 (strongly agree/always). The Greek version was utilized, with Cronbach’s alpha values of 0.878 for the total scale, and 0.791, 0.786, and 0.848 for the subscales, respectively [34].

Work engagement was assessed using the Utrecht Work Engagement Scale-3 (UWES-3), which consists of three items rated on a seven-point Likert scale (0 = never to 6 = every day) [35]. Higher average scores indicate greater engagement. The Greek version was used, with a Cronbach’s alpha of 0.825 [36].

Demographic information collected included gender (female or male), age (as a continuous variable), educational attainment (high school graduate, university graduate or MSc/PhD diploma), and work experience (in years).

### 2.3. Ethical Considerations

The study was conducted in line with the Declaration of Helsinki and received approval from the Ethics Committee of the Faculty of Nursing, National and Kapodistrian University of Athens (approval number 15, 9 December 2024) [37]. Participation was anonymous and voluntary, with all participants receiving information about the study’s purpose and design and providing informed consent.

### 2.4. Statistical Analysis

Categorical variables were summarized as counts and percentages, while continuous variables were described using the mean, standard deviation, median, and interquartile range. The Kolmogorov–Smirnov test and Q-Q plots were used to assess the normality of continuous variables, which were found to be normally distributed. Workplace gaslighting was analyzed as the independent variable, with anxiety, depression, quiet quitting, and work engagement as dependent variables. Demographic characteristics were treated as potential confounders. Both simple and multivariable linear regression analyses were performed to explore associations between variables. Initially, simple linear regression was conducted, followed by multivariable modeling, with confounders removed stepwise to assess the independent effect of gaslighting. Due to high correlations between the two GWS factors (“loss of self-trust” and “abuse of power”; Pearson’s r = 0.806, *p* < 0.001) and between age and work experience (Pearson’s r = 0.883, *p* < 0.001), only the total GWS score and work experience (not age) were included in the final multivariable models to avoid multicollinearity. Results are reported as unadjusted and adjusted beta coefficients, 95% confidence intervals, and *p*-values, with significance set at *p* < 0.05. All analyses were conducted using IBM SPSS Statistics for Windows, Version 28.0.

## 3. Results

### 3.1. Demographic Characteristics

The demographic details of the participating workers are presented in Table 1. A total of 291 workers took part in the study, with the majority being female (77%). The average age was 42.15 years (SD = 10.28), and the median age was 43 years (interquartile range = 14). More than half of the workers (55.3%) held a postgraduate degree (MSc or PhD). The mean duration of work experience among participants was 16.69 years (SD = 9.48), with a median of 17 years (interquartile range = 14).

### 3.2. Study Scales

Descriptive statistics for the main study measures are summarized in Table 2. The average score on the Gaslighting at Work Scale (GWS) was 2.66 (SD = 1.08), indicating a moderate presence of gaslighting behaviors from supervisors. Among the GWS subscales, “abuse of power” (mean = 2.66) was reported more frequently than “loss of self-trust” (mean = 2.09).

Regarding mental health, workers reported moderate levels of anxiety (mean = 2.67) and depression (mean = 2.63). The mean score for quiet quitting was 2.41, also reflecting moderate levels. Of the quiet quitting subscales, “lack of motivation” was most prominent (mean = 2.70), followed by “lack of initiative” (mean = 2.39) and “detachment” (mean = 2.28). Work engagement, as measured by the UWES-3, was moderate with a mean of 3.62 (SD = 1.60).

### 3.3. Association Between Workplace Gaslighting, Anxiety, and Depression

Our analysis showed that higher levels of workplace gaslighting were significantly associated with increased anxiety and depression among workers (see Table 3). After controlling for gender, education, and work experience, the GWS score remained a significant predictor for both anxiety (b = 0.997, 95% CI: 0.835–1.160, *p* < 0.001) and depression (b = 1.028, 95% CI: 0.858–1.197, *p* < 0.001). In other words, workers who experienced more gaslighting from supervisors also reported higher levels of anxiety and depressive symptoms.

### 3.4. Association Between Workplace Gaslighting, Quiet Quitting, and Work Engagement

Table 4 presents the results for quiet quitting and work engagement. After adjusting for the confounding variables, a higher GWS score was positively associated with quiet quitting (β = 0.362, 95% CI: 0.217–0.458, *p* < 0.001) and negatively associated with work engagement (β = −0.373, 95% CI: −0.486 to −0.266, *p* < 0.001). This indicates that workers who experienced more gaslighting at work were more likely to engage in quiet quitting behaviors and less likely to feel engaged in their work.

## 4. Discussion

The present study demonstrated that employees experience a moderate level of gaslighting, which was found to be a significant predictor of anxiety, depression, quiet quitting, and work engagement. Our findings cannot be directly compared with those of other studies, as the literature on workplace gaslighting remains extremely limited. To the best of our knowledge, only one study has previously examined the impact of gaslighting on nursing staff, and our results are consistent with those reported in that study. Specifically, gaslighting was found to be associated with increased levels of depression and anxiety, to reinforce quiet quitting behaviors, and to concurrently reduce work engagement [22]. In contrast to the workplace setting, gaslighting has primarily been studied within the context of interpersonal relationships, where its impact on the deterioration of victims’ mental health has been well documented [38,39,40].

Employees often find themselves trapped in a peculiar vise, where on one side they face intense pressure due to increased job demands and limited resources, while on the other, they are subjected to abusive behavior from a management that should ideally serve as their primary ally and source of support. As a result, employees experience significant physical and mental health problems [41,42,43]. Also, survivors’ outcomes encompass alterations in perception and memory, heightened emotional distress, increased social withdrawal, and the use of various resistance strategies [44]. This withdrawal may lead to, or further reinforce, employee disengagement and the adoption of quiet quitting.

When employees’ mental health deteriorates, their work engagement declines, while their tendency toward quiet quitting increases [45,46]. However, the consequences also extend to organizations. When employees engage in quiet quitting, they are more likely to exhibit turnover intention [47]. Turnover intention, which essentially reflects actual turnover behavior [48], entails a substantial economic cost for the organization, encompassing the processes of employee recruitment, training, and adaptation to the new position. In addition, it imposes a non-economic cost related to the quality of the services provided and the level of customer satisfaction [49]. Moreover, low levels of work engagement are associated with reduced job performance and diminished innovative behavior [50]. Work engagement, when accompanied by supervisory support, can negatively influence employees’ turnover intention [51].

Leadership support has emerged as a critical determinant in safeguarding employees’ mental health [52]. Through behaviors such as emotional support, practical assistance, role modeling, stigma reduction, recognition of warning signs, and appropriate response to them, supervisors can effectively protect and promote the psychological well-being of their staff [53]. Moreover, when leaders foster strengthening, connecting, empowering, and inspiring behaviors, they mitigate quiet quitting and enhance employees’ work engagement [14].

The protection of employees from gaslighting behaviors constitutes a core responsibility of senior management. The essential step in addressing such behaviors is the adoption of a zero-tolerance policy, coupled with the encouragement and empowerment of employees to promptly report any experiences of abusive conduct. However, gaslighting can also manifest as a form of “silent abuse,” which is often difficult to detect or expose. Employees subjected to psychological manipulation may either fail to recognize themselves as victims (due to the distortion of their judgment) or may choose silence as a coping mechanism, particularly when experiencing exhaustion resulting from the ongoing abuse [54]. The implementation of validated assessment tools for identifying abusive behaviors could serve as an effective organizational strategy to uncover and address such harmful dynamics.

Our study had several limitations. Primarily, the cross-sectional design of our study did not allow for the determination of causal relationships among workplace gaslighting, mental health, quiet quitting, and work engagement. Longitudinal studies should be conducted by reporting employees’ attitudes over time to further validate our findings. Moreover, although the required minimum sample size was achieved, the use of convenience sampling may have introduced selection bias. Furthermore, the reliance on an online survey for participant recruitment likely resulted in self-selection bias. Thus, scholars should conduct studies with random, stratified and representative samples of employees to establish more solid results. Moreover, since our study was carried out in a European setting, further investigations across different cultural contexts are required to deepen the understanding of the relationships among the study variables. Another limitation of this study was that we did not measure anxiety or depression disorders among our participants. Therefore, we cannot compare our findings across individuals with anxiety or depression disorders and healthy individuals. Since this information could provide invaluable information, scholars in future studies could differentiate their results according to anxiety or depression status. We should recognize that although we eliminated several confounding factors, several other factors could affect the association between workplace gaslighting, anxiety, depression, quiet quitting, and work engagement. For instance, resilience is a crucial factor that could interact between these variables either as a confounder or as a mediator. Resilience may mitigate the impact of workplace gaslighting on the employees’ mental health and work life. Future studies should examine in depth the role of characteristics such as resilience.

## 5. Conclusions

Employees are exposed to demanding working conditions on a daily basis, aiming to enhance both their effectiveness and the overall development of the organization in which they work. Although supervisors bear the responsibility and hold the role of supporting their staff, employees are often subjected to abusive behaviors. Within the framework of the present study, gaslighting was found to adversely affect employees’ mental health while simultaneously leading to quiet quitting and reduced work engagement. These consequences impact both the employees and the organization as a whole. The findings of the present study, as well as those of other investigations on abusive behaviors in the workplace, should be disseminated not only through scientific journals and conferences, but also to relevant stakeholders (e.g., organizational leadership). Such dissemination is essential to ensure that they are informed about the existence and magnitude of these phenomena and can adopt zero-tolerance policies and corresponding actions. Senior management is therefore urged to adopt a zero-tolerance approach toward such behaviors and to empower employees to report them without fear of retaliation. In many cases, gaslighting can be a “silent” phenomenon that remains unnoticed by other employees. Therefore, the establishment of formal procedures and the encouragement of staff to report such incidents are essential components of senior management’s efforts to protect employees and to ensure a healthy and safe work environment. Employees, for their part, should not hesitate to report such behaviors to the senior management of their organizations through official procedures. In parallel, they may seek psychological support within their workplace from specialized professionals, as well as from psychosocial support services (public or NGO-based). Such support structures are available in Greece and are funded by the State, the Regional Authorities, and the European Union.

## Figures and Tables

**Table 1:** Demographic characteristics of workers (N = 291).

Characteristic	N	%
Gender		
Males	67	23.0
Females	224	77.0
Age (years) ^a^	42.15	10.28
Educational attainment		
High school graduate	25	8.6
University graduate	105	36.1
MSc/PhD diploma	161	55.3
Work experience (years) ^a^	16.69	9.48

^a^ mean, standard deviation.

**Table 2:** Descriptive statistics for the study scales (N = 291).

Scale	Mean	Standard Deviation	Median	Interquartile Range
Gaslighting at Work Scale	2.66	1.08	2.50	1.83
Loss of self-trust	2.09	1.00	2.00	1.40
Abuse of power	2.66	1.08	2.50	1.83
Patient Health Questionnaire-4	5.31	3.35	5.00	5.00
Anxiety	2.67	1.75	2.00	3.00
Depression	2.63	1.78	2.00	2.00
Quiet Quitting Scale	2.41	0.79	2.33	0.89
Detachment	2.28	0.87	2.25	1.00
Lack of initiative	2.39	0.94	2.33	1.33
Lack of motivation	2.70	1.02	2.50	1.00
Utrecht Work Engagement Scale-3	3.62	1.60	3.67	2.67

**Table 3:** Linear regression models with anxiety and depression as dependent variables and workplace gaslighting as the independent variable (N = 291).

Dependent Variable	Unadjusted b	95% CI	*p*-Value	Adjusted b	95% CI	*p*-Value
Anxiety ^a^	1.028	0.862–1.194	<0.001	0.997	0.835–1.160	<0.001
Depression ^b^	1.038	0.868–1.208	<0.001	1.028	0.858–1.197	<0.001

^a^ For the final multivariable model: R^2^ = 0.382, *p* < 0.001. ^b^ For the final multivariable model: R^2^ = 0.353, *p* < 0.001. All models are adjusted for gender, educational attainment, and work experience. CI: confidence interval.

**Table 4:** Linear regression models with quiet quitting and work engagement as dependent variables and workplace gaslighting as the independent variable (N = 291).

Dependent Variable	Unadjusted b	95% CI	*p*-Value	Adjusted b	95% CI	*p*-Value
Quiet quitting ^a^	0.272	0.185–0.358	<0.001	0.271	0.186 to 0.357	<0.001
Work engagement ^b^	−0.609	−0.781–−0.436	<0.001	−0.601	−0.771 to −0.431	<0.001

^a^ For the final multivariable model: R^2^ = 0.150, *p* < 0.001. ^b^ For the final multivariable model: R^2^ = 0.190, *p* < 0.001. All models are adjusted for gender, educational attainment, and work experience. CI: confidence interval.

## Data Availability

The data used in this study are openly available in Figshare at https://doi.org/10.6084/m9.figshare.29094980.v1 (accessed on 10 October 2025).

## References

[1] Xu Y., Tan T.F., Netessine S. (2022). The Impact of Workload on Operational Risk: Evidence from a Commercial Bank. Manag. Sci..

[2] Elliott K.C., Lincoln J.M., Flynn M.A., Levin J.L., Smidt M., Dzugan J., Ramos A.K. (2022). Working Hours, Sleep, and Fatigue in the Agriculture, Forestry, and Fishing Sector: A Scoping Review. Am. J. Ind. Med..

[3] Rokhim R., Takwin B., Basrowi R.W., Soemarko D.S., Rahadian A., Ekowati M., Samah K., Moeloek N.D.F. (2025). Fatigue and Lack of Vigor’s as a Frequent Work Stress among Financial Workers in Indonesia. Front. Public Health.

[4] Mannocci A., Marchini L., Scognamiglio A., Sinopoli A., De Sio S., Sernia S., La Torre G. (2018). Are Bank Employees Stressed? Job Perception and Positivity in the Banking Sector: An Italian Observational Study. Int. J. Environ. Res. Public Health.

[5] Koutsimani P., Montgomery A., Georganta K. (2019). The Relationship Between Burnout, Depression, and Anxiety: A Systematic Review and Meta-Analysis. Front. Psychol..

[6] Giorgi G., Arcangeli G., Perminiene M., Lorini C., Ariza-Montes A., Fiz-Perez J., Di Fabio A., Mucci N. (2017). Work-Related Stress in the Banking Sector: A Review of Incidence, Correlated Factors, and Major Consequences. Front. Psychol..

[7] Teh Z.Y., Yusoff M.Z. (2025). The Impact of Workload and Job Satisfaction on Employee Intention to Leave in the Logistics Industry: A Study of Small and Medium Enterprises (SMEs) in Selangor, Malaysia. Res. Manag. Technol. Bus..

[8] Moisoglou I., Katsiroumpa A., Katsapi A., Konstantakopoulou O., Galanis P. (2025). Poor Nurses’ Work Environment Increases Quiet Quitting and Reduces Work Engagement: A Cross-Sectional Study in Greece. Nurs. Rep..

[9] Galanis P., Moisoglou I., Katsiroumpa A., Gallos P., Kalogeropoulou M., Meimeti E., Vraka I. (2025). Workload Increases Nurses’ Quiet Quitting, Turnover Intention, and Job Burnout: Evidence from Greece. AIMS Public Health.

[10] Ng T.W.H. (2017). Transformational Leadership and Performance Outcomes: Analyses of Multiple Mediation Pathways. Leadersh. Q..

[11] Jensen U.T., Bro L.L. (2018). How Transformational Leadership Supports Intrinsic Motivation and Public Service Motivation: The Mediating Role of Basic Need Satisfaction. Am. Rev. Public Adm..

[12] Khalili A. (2016). Linking Transformational Leadership, Creativity, Innovation, and Innovation-Supportive Climate. Manag. Decis..

[13] Montano D., Schleu J.E., Hüffmeier J. (2023). A Meta-Analysis of the Relative Contribution of Leadership Styles to Followers’ Mental Health. J. Leadersh. Organ. Stud..

[14] Moisoglou I., Katsiroumpa A., Papathanasiou I.V., Konstantakopoulou O., Katharaki M., Malliarou M., Tsaras K., Prasini I., Rekleiti M., Galanis P. (2025). Engaging Leadership Reduces Quiet Quitting and Improves Work Engagement: Evidence from Nurses in Greece. Nurs. Rep..

[15] Stern R. (2007). The Gaslight Effect: How to Spot and Survive the Hidden Manipulation Others Use to Control Your Life.

[16] Darke L., Paterson H., van Golde C. (2025). Illuminating Gaslighting: A Comprehensive Interdisciplinary Review of Gaslighting Literature. J. Fam. Viol..

[17] Abramson K. (2014). Turning up the Lights on Gaslighting. Philos. Perspect..

[18] Klein W., Wood S., Bartz J. (2023). A Historical Review of Gaslighting: Tracing Changing Conceptualizations Within Psychiatry and Psychology. PsyArXiv.

[19] Sweet P.L. (2019). The Sociology of Gaslighting. Am. Sociol. Rev..

[20] Clark C.M. (2024). Navigating the Challenging Complexities of Gaslighting in Nursing Academe. Teach. Learn. Nurs..

[21] Moisoglou I., Katsiroumpa A., Konstantakopoulou O., Papathanasiou I.V., Katsapi A., Prasini I., Chatzi M., Galanis P. (2025). Workplace Gaslighting Is Associated with Nurses’ Job Burnout and Turnover Intention in Greece. Healthcare.

[22] Katsiroumpa A., Moisoglou I., Konstantakopoulou O., Gallos P., Rekleiti M., Rizos F., Galanis P. (2025). Workplace Gaslighting Affects Nurses’ Mental Health and Work Life: Evidence from Greece. Preprints.

[23] Farid H., Zhang Y., Tian M., Lu S. (2024). Unmasking the Interplay between Gaslighting and Job Embeddedness: The Critical Roles of Coworker Support and Work Motivation. J. Manag. Organ..

[24] Fulcher C., Ashkanasy N.M., Troth A.C., Ashkanasy N.M., Humphrey R.H. (2023). Supervisory Gaslighting and Its Effects on Employee Affective Commitment. Emotions During Times of Disruption.

[25] Klein W., Li S., Wood S. (2023). A Qualitative Analysis of Gaslighting in Romantic Relationships. Pers. Relatsh..

[26] Tepper B.J. (2000). Consequences of Abusive Supervision. Acad. Manag. J..

[27] Martinko M.J., Harvey P., Brees J.R., Mackey J. (2013). A Review of Abusive Supervision Research. J. Organ. Behav..

[28] Fatima T., Majeed M., Shah S.Z.A. (2018). Jeopardies of Aversive Leadership: A Conservation of Resources Theory Approach. Front. Psychol..

[29] von Elm E., Altman D.G., Egger M., Pocock S.J., Gøtzsche P.C., Vandenbroucke J.P. (2007). The Strengthening the Reporting of Observational Studies in Epidemiology (STROBE) Statement: Guidelines for Reporting Observational Studies. Lancet.

[30] Katsiroumpa A., Moisoglou I., Konstantakopoulou O., Tsiachri M., Kolisiati A., Galanis P. (2025). The Gaslighting at Work Scale: Development and Initial Validation. J. Workplace Behav. Health.

[31] Kroenke K., Spitzer R.L., Williams J.B.W., Löwe B. (2009). An Ultra-Brief Screening Scale for Anxiety and Depression: The PHQ–4. Psychosomatics.

[32] Karekla M., Pilipenko N., Feldman J. (2012). Patient Health Questionnaire: Greek Language Validation and Subscale Factor Structure. Compr. Psychiatry.

[33] Galanis P., Katsiroumpa A., Vraka I., Siskou O., Konstantakopoulou O., Moisoglou I., Gallos P., Kaitelidou D. (2023). The Quiet Quitting Scale: Development and Initial Validation. AIMS Public Health.

[34] Galanis P., Katsiroumpa A., Vraka I., Siskou O., Konstantakopoulou O., Katsoulas T., Moisoglou I., Gallos P., Kaitelidou D. (2024). Nurses Quietly Quit Their Job More Often than Other Healthcare Workers: An Alarming Issue for Healthcare Services. Int. Nurs. Rev..

[35] Schaufeli W.B., Shimazu A., Hakanen J., Salanova M., De Witte H. (2019). An Ultra-Short Measure for Work Engagement. Eur. J. Psychol. Assess..

[36] Katsiroumpa A., Moisoglou I., Kalogeropoulou M., Konstantakopoulou O., Gallos P., Tsiachri M., Galanis P. (2024). Utrecht Work Engagement Scale (3 Items Version): Translation and Validation in Greek. Int. J. Caring Sci..

[37] World Medical Association (2013). World Medical Association Declaration of Helsinki: Ethical Principles for Medical Research Involving Human Subjects. JAMA.

[38] Imtiaz A., Javed A., Qureshi A. (2025). Gaslighting, Emotional Abuse, and Mental Health in Adults’ Romantic Relationships. J. Health Wellness Community Res..

[39] Ciabatti M., Nerini A., Matera C. (2025). Gaslighting Experience, Psychological Health, and Well-Being: The Role of Self-Compassion and Social Support. J. Interpers. Violence.

[40] Thulin E.J., Heinze J.E. (2025). Gaslighting in Teen Dating Violence: Links to Anxiety and Depression. J. Interpers. Violence.

[41] Upadyaya K., Vartiainen M., Salmela-Aro K. (2016). From Job Demands and Resources to Work Engagement, Burnout, Life Satisfaction, Depressive Symptoms, and Occupational Health. Burn. Res..

[42] Santos C., Coelho A., Filipe A., Marques A.M.A. (2023). The Dark Side of Leadership: Abusive Supervision and Its Effects on Employee’s Behavior and Well-Being. J. Strategy Manag..

[43] Lu J., Yu Y., Zhao Y., Jenkin M. (2021). The Correlation between Workers’ Working Pressure and Physical and Mental Health Analyzed by the Job Demand-Resource Stress Model. Work.

[44] Adair J. (2025). Defining Gaslighting in Gender-Based Violence: A Mixed-Methods Systematic Review. Trauma Violence Abus..

[45] Geng R., Geng X., Geng S. (2025). Identifying Key Antecedents of Quiet Quitting Among Nurses: A Cross-Profession Meta-Analytic Review. J. Adv. Nurs..

[46] Veromaa V., Kautiainen H., Korhonen P.E. (2017). Physical and Mental Health Factors Associated with Work Engagement among Finnish Female Municipal Employees: A Cross-Sectional Study. BMJ Open.

[47] Galanis P., Moisoglou I., Malliarou M., Papathanasiou I.V., Katsiroumpa A., Vraka I., Siskou O., Konstantakopoulou O., Kaitelidou D. (2024). Quiet Quitting among Nurses Increases Their Turnover Intention: Evidence from Greece in the Post-COVID-19 Era. Healthcare.

[48] Wong K.F.E., Cheng C. (2020). The Turnover Intention–Behaviour Link: A Culture-Moderated Meta-Analysis. J. Manag. Stud..

[49] Bae S.-H. (2022). Noneconomic and Economic Impacts of Nurse Turnover in Hospitals: A Systematic Review. Int. Nurs. Rev..

[50] Kim W., Kolb J.A., Kim T. (2013). The Relationship Between Work Engagement and Performance: A Review of Empirical Literature and a Proposed Research Agenda. Hum. Resour. Dev. Rev..

[51] Pattnaik S.C., Panda N. (2020). Supervisor Support, Work Engagement and Turnover Intentions: Evidence from Indian Call Centres. J. Asia Bus. Stud..

[52] Wu A., Roemer E.C., Kent K.B., Ballard D.W., Goetzel R.Z. (2021). Organizational Best Practices Supporting Mental Health in the Workplace. J. Occup. Environ. Med..

[53] Hammer L.B., Dimoff J., Mohr C.D., Allen S.J. (2024). A Framework for Protecting and Promoting Employee Mental Health through Supervisor Supportive Behaviors. Occup. Health Sci..

[54] Xu A.J., Loi R., Lam L.W. (2015). The Bad Boss Takes It All: How Abusive Supervision and Leader–Member Exchange Interact to Influence Employee Silence. Leadersh. Q..

